# Role of Morbidity Clusters in Midlife on Ischemic Stroke Incidence and Severity: The ARIC Study

**DOI:** 10.1161/STROKEAHA.124.049496

**Published:** 2025-08-20

**Authors:** Marco Egle, Renee C. Groechel, Michelle C. Johansen, Anna M. Kucharska-Newton, Rebecca F. Gottesman, Silvia Koton

**Affiliations:** Stroke Branch, National Institute of Neurological Disorders and Stroke, Intramural Research Program, National Institutes of Health, Bethesda, MD (M.E., R.C.G., R.F.G.).; Department of Neurology, The Johns Hopkins University School of Medicine, Baltimore, MD (M.C.J.).; Department of Epidemiology, University of North Carolina at Chapel Hill Gillings School of Global Public Health (A.M.K.-N.).; Department of Epidemiology, University of Kentucky, Lexington (A.M.K.-N.).; Department of Nursing, The Stanley Steyer School of Health Professions, Tel Aviv University, Israel (S.K.).; Department of Epidemiology, Johns Hopkins University School of Public Health, Baltimore, MD (S.K.).

**Keywords:** atherosclerosis, atrial fibrillation, hypertension, ischemic stroke, stroke

## Abstract

**BACKGROUND:**

There is a strong association between vascular risk factors, particularly in midlife, and stroke risk; therefore, the co-occurrence of multiple risk factors may be especially informative. This study used a machine-learning–based cluster analysis to group individuals into clusters based on similar clinical profiles in midlife and assessed the clusters’ associations with stroke risk and severity.

**METHODS:**

Participants (N=15 404) without prevalent stroke from the ARIC study (Atherosclerosis Risk in Communities) were included. An unsupervised agglomerative hierarchical clustering approach was used to allocate participants into clusters based on the presence of clinical risk factors in midlife: hypertension, diabetes, coronary heart disease, heart failure, atrial fibrillation, renal dysfunction, and peripheral artery disease. Clusters were then characterized by their defining features. In Cox proportional hazard models, the association of the clusters with overall stroke incidence (and with ischemic stroke incidence stratified by stroke severity) was tested. Multinomial logistic regression models were used to examine the association of morbidity clusters with outcomes of no stroke, stroke before the age of 70 years, and stroke at age of ≥70 years.

**RESULTS:**

Of 1424 incident ischemic strokes diagnosed from baseline (1987–1989) to December 31, 2020, 1104 included National Institutes of Health Stroke Scale (NIHSS) grading (minor-mild stroke: NIHSS score ≤5 [n=687]; moderate-severe stroke: NIHSS score >5 [n=417]). The cluster analysis identified 9 distinct clusters in the population with defining features: cluster 1 (relatively healthy); cluster 2 (smoking); cluster 3 (cancer); cluster 4 (peripheral artery disease); cluster 5 (obesity, diabetes, hypertension, and hypertriglyceridemia); cluster 6 (coronary heart disease); cluster 7 (atrial fibrillation); cluster 8 (heart failure); and cluster 9 (renal dysfunction). Compared with cluster 1, clusters 2 to 9 were each associated with a greater stroke risk, with the largest effect estimate for cluster 9 (hazard ratio, 3.00 [95% CI, 2.00–4.50]). The association with moderate-severe stroke incidence (versus no stroke) was also strongest for cluster 9 (hazard ratio, 4.78 [95% CI, 2.62–8.74]). Except for cluster 5 (which was associated with stroke at any age), all midlife morbidity clusters were associated with greater stroke risk before the age of 70 years but not after the age of 70 years.

**CONCLUSIONS:**

The findings emphasize the importance of morbidity clusters in midlife for stroke incidence and severity.

Stroke is the second leading cause of mortality and the leading cause of disability in adults worldwide.^1^ One in 4 individuals experiences a stroke in their lifetime, and the ischemic type accounts for ≈85% of all cases. Up to 90% of ischemic stroke cases have been shown to be attributable to modifiable risk factors, including hypertension, smoking, alcohol abuse, hyperlipidemia, physical inactivity, diabetes, and smoking.^2^ The etiologic relation of the risk factors with ischemic stroke varies by subtype, and different subtypes frequently result in different stroke severities.^3,4^ Addressing those risk factors is crucial to mitigate the global increase in stroke events and the strong rise of stroke-related disability and mortality, particularly in medium and low-income countries.^1^

**See related article, p 2942**

When evaluating the importance of risk factors in stroke, it is crucial to consider when in the life course risk is being evaluated, and how the risk of multiple frequently co-occurring risk factors and comorbidities is being considered.^5^ Studies have suggested that there may be an age-dependent effect of risk factors, with the greatest relative risk of stroke seen for risk factors that are present in midlife.^6,7^ Furthermore, composite risk scores have been developed to capture the importance of comorbidity in evaluating one’s stroke risk,^8^ based on presumptive clinical risk of co-occurring morbidities. Another approach to considering the overall comorbidity burden is to cluster similar individuals together, entirely driven by individual-based similarities and differences and not based on clinical hypotheses about co-occurring risk factors. This approach can be conducted, as demonstrated by multiple research studies in the literature,^9–13^ by using unsupervised machine-learning methods to generate these clusters, as a way to account for the multiplicative adverse interactions of co-occurring diseases.^9,14^ The prognostic clinical value of this approach has, for instance, been demonstrated by Vetrano et al,^9^ showing that clustered subgroups characterized by distinct multimorbid patterns had markedly different mortality risks over a 12-year period.

This study employs a similar methodology using a hierarchical clustering algorithm to identify clustered subgroups of individuals with shared morbidities in their midlife in the ARIC study (Atherosclerosis Risk in Communities) and assesses whether derived clusters of morbidities are also valuable to predict greater lifetime risk for ischemic stroke. We furthermore hypothesized that the associations of the midlife clusters with moderate-severe stroke will be stronger than with minor-mild stroke incidence versus no stroke. We also tested whether the associations differ by demographics. Finally, we explored the clusters’ midlife risk profiles in predicting midlife (age, <70 years) versus late-life stroke (age, ≥70 years). Age >70 years has previously been used as a cut point to define late-life stroke.^15^

## Methods

The study was approved by the institutional review boards at all associated institutions, and informed consent was obtained for all participants at each visit. The data used for this analysis are available per ARIC policies. The study followed the STROBE (Strengthening the Reporting of Observational Studies in Epidemiology) reporting guideline. The data are available through public databases.

### Ethical Approval and Consent

The study was performed in accordance with the ethical standards as laid down in the 1964 Declaration of Helsinki and its later amendments or comparable ethical standards.

### Study Population

The ARIC study is an ongoing community-based prospective study of 15 792 participants aged 45 to 64 years at baseline (visit 1; 1987–1989) recruited from 4 diverse US communities. The present study uses data from in-person visits, which were visits 1 to 7, and from a primarily telephone-based visit, which was visit 8 (2020).^16^ There has also been ongoing surveillance between study visits where individuals have been contacted by telephone annually in earlier years, and more recently semiannually, with hospitalization record abstraction and adjudication of cardiovascular events, followed by informant interviews after a participant’s death. Information about mortality and dates of death is tracked throughout the cohorts. Race was self-reported at baseline. Individuals who were not Black or White, and those who were not White participants at Minneapolis or Maryland (n=103), were excluded from this analysis given the small race-center numbers. Participants were furthermore excluded if they reported a stroke (n=282) or had unknown stroke status (n=3) before baseline, resulting in a sample size of 15 404 participants (Figure S1).

### Stroke Incidence

Information on clinical stroke over study follow-up through the year 2020 was ascertained based on hospital record reviews and through annual/semiannual phone interviews. Potential stroke-related admission was furthermore identified through hospital discharge data.^17^ Stroke-related death cases were identified through linkage with the National Death Index. Classification by stroke subtype as ischemic or hemorrhagic was performed independently by computer-generated algorithms and a study physician, with disagreement between the 2 adjudicated by another physician. The definitions for stroke subtypes and the standardized criteria have remained consistent across the follow-up period. Definite stroke events were based on autopsy or imaging confirmation; probable stroke events lacked exclusionary findings on imaging and had an appropriate clinical presentation.^18^ Adjudicated definite or probable ischemic strokes occurring after baseline were classified in this study as incident ischemic strokes.

### Stroke Severity

Stroke severity data were abstracted by trained physicians from available stroke hospitalization records between 1987 to 2019 for events defined in ARIC as definite or probable ischemic stroke.^19^ Stroke severity as defined by the National Institutes of Health Stroke Scale (NIHSS) was determined using an algorithm suitable for hospital chart-based evaluation of NIHSS scores.^20^ For this study, stroke severity was considered in 2 categories: minor-mild (NIHSS score ≤5) and moderate-severe (NIHSS score >5).

### Morbidities and Mortality

Information about risk factors and morbidities was obtained at baseline (visit 1; age, 45–64 years), unless otherwise specified below. Vascular risk factors were selected for the analyses based on being consistently measured over follow-up and being hypothesized to be important in stroke risk. Measurements of blood pressure were performed 3×; hypertension was present if the mean of the last 2 measurements was >140 mm Hg (systolic) or >90 mm Hg (diastolic), or if the participant took antihypertensive medication. Participants were classified as having diabetes when (1) showing a fasting glucose level ≥126 mg/dL, (2) showing a nonfasting glucose level ≥200 mg/dL, (3) reporting to be diagnosed with diabetes by a physician, or (4) using oral diabetes medications or insulin. Prevalent coronary heart disease (CHD) was based on indications of previous myocardial infarctions (by adjudicated ECG data at rest or by self-reported physician diagnosis) or cardiovascular revascularizations. Prevalent heart failure (HF) was identified using the Gothenburg criteria and the review of medication use.^21^ A diagnosis of atrial fibrillation was ascertained from the ECG recording at baseline.^22^ Estimated glomerular filtration rate was computed with the Chronic Kidney Disease Epidemiology Collaboration equation using age, sex, race, and measured serum creatinine as input information.^23^ Individuals were classified as having renal dysfunction with an estimated glomerular filtration rate <60 mL/(min·1.73 m^2^).^24^ Ankle-brachial index ≤0.9 was used to determine lower-extremity peripheral artery disease. Hypercholesterolemia and hypertriglyceridemia were defined as total cholesterol ≥240 mg/dL and as total triglycerides ≥177 mg/dL, respectively. Obesity was defined as a body mass index ≥30 kg/m^2^. Symptoms of vital exhaustion were measured using the Maastricht questionnaire at visit 2 (age, 48–67 years), with a score >13 defined as consistent with vital exhaustion.^25^ The 21-item questionnaire assesses domains such as physical and mental state of excessive fatigue, hopelessness, demoralization, and irritability. Vital exhaustion has been considered a proxy for depression, and evidence has shown that individuals with vital exhaustion have an increased risk of adverse cardiac events, stroke, and all-cause mortality.^26,27^ Information about current smoking, current drinking, and having ever been diagnosed with cancer was self-reported.

### Demographic Measures

Demographics on age, sex, education, and self-reported race were collected at visit 1. Due to varying proportions of racial groups across the 4 ARIC sites, race and center were combined into 1 variable.

### Statistical Analysis

Multiple imputation (m=10, maxit=15) by chained equation (MICE) with mode aggregation was used to impute missing data for education (n=23), CHD (n=331), current drinking (n=67), current smoking (n=12), diabetes (n=142), HF (n=273), hypertension (n=77), peripheral artery disease (n=574), cancer (n=53), atrial fibrillation (n=193), hypercholesterolemia (n=239), hypertriglyceridemia (n=236), obesity (n=24), renal dysfunction (n=145), and vital exhaustion (n=1475).^28,29^ The average proportion of missing data was only 1.8%; MICE was performed operating under the Missing at Random assumption.

A multiple correspondence analysis with a singular value decomposition technique was first used as a dimensionality reduction approach to reduce the number of space dimensions and to project the observations on these newly created dimensions with their corresponding coordinates. Over the years, multiple correspondence analysis has become a critical tool to determine relationships between multiple categorical variables. This approach allowed us to focus on a limited number of uncorrelated variables, that is, dimensions (n=13), while capturing ≈95% of the data’s variation. We chose the a priori 95% threshold to account for the high-quality cohort data and to filter out potential noise that could influence the cluster computation. The Euclidean distance was then used on the transformed data structure to quantify the similarity in the coordinates between the individuals. We then used an unsupervised hierarchical cluster analysis using Ward’s agglomerative criterion to assign individuals to more homogenous groups characterized by similar clinical profiles^30^ (HCPC function, from FactoMineR package; Vienna, Austria). The optimal number of clusters was determined based on the gain of the within-inertia (variance within the clusters). K-means consolidation was additionally used to make the clusters’ partition more robust. The clusters were then characterized by the risk factors using the observed/expected ratio and the marginal proportion of a condition. Risk factors were labeled as defining features when both the observed-to-expected ratio for that risk factor within a given cluster was ≥1.5 and the marginal proportion of a condition was ≥25%.^10^ A graphical overview of the cluster analysis described here is provided in Figure S2. A graphical depiction of the statistical analysis focusing on the critical multiple correspondence analysis steps and the hierarchical cluster analysis can be found in Figure S3. It is worth emphasizing that the hierarchical cluster analysis is part of the unsupervised machine-learning family and therefore different from supervised machine-learning models that are primarily used for predictions (Table S1).

Descriptive statistics were computed to evaluate cluster-related differences in demographics. Cox proportional hazard models were used to test the associations of the clusters with stroke incidence and stroke severity (minor-mild stroke: NIHSS score ≤5; moderate-severe stroke: NIHSS score >5, each compared with absence of stroke), while accounting for age, sex, education, and race-center. The cluster with no defined morbidities, the healthiest cluster group with an unspecific comorbidity burden, was the reference group. The underlying proportional hazard assumptions, checked by graphical inspection of the scaled Schoenfeld residuals and the log-minus-log plot, showed no evidence of violations. The risk of an inflated type-1 error rate was low since the risk factors underlying the clusters have all been shown to be associated with stroke risk in prior literature. The interaction effects between the clusters and sex, education, and race, each, were tested in separate regression models. In a subsequent sensitivity analysis, a competing risk model based on the Fine-Gray method was used to test whether the associations remained significant when accounting for mortality as a competing event. To better understand the influence of mortality on the mid- to late-life cluster trajectories, an alluvial plot was created that visualizes the proportion of participants in each cluster who, by the age of 70 years, had either experienced a stroke, who were alive having not experienced a stroke, and who were deceased without stroke before the age of 70 years.

Using a multinomial logistic regression model, the association between the clusters and the age of stroke was tested while accounting for baseline age, sex, education, and race-center. The multinomial dependent variable consisted of 3 factor levels: (1) stroke incidence before the age of 70 years, (2) stroke incidence at or after the age of 70 years, and (3) no stroke incidence (reference outcome category).

## Results

The median (Q1, Q3) age of the cohort at baseline was 54 (49, 59) years, and most participants were women (55%) and had at least a high school education (76%; Table 1). One quarter of participants were of Black race (26%). Between baseline and December 31, 2020, 1424 (9.3%) participants experienced an ischemic stroke. NIHSS grading was available for 1104 individuals between baseline and 2019, of whom 417 (38%) experienced a moderate-severe stroke.

**Table 1.**
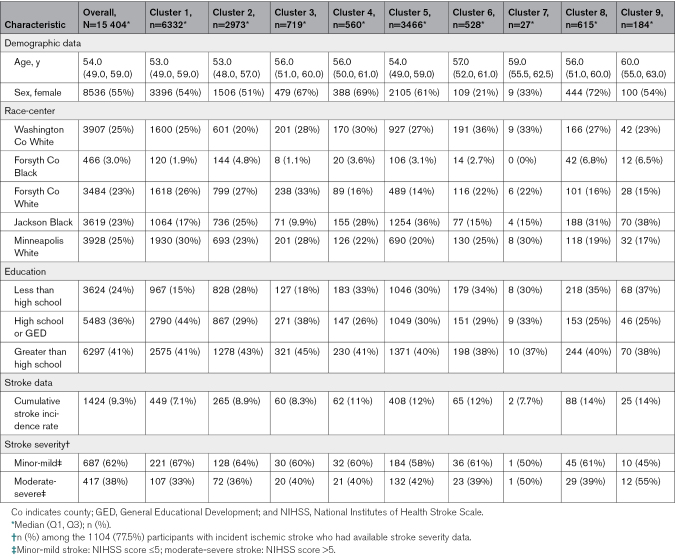
Demographic and Stroke-Related Differences Between the Clusters

### Cluster Analysis

The algorithm divided the cohort data into 9 clusters with varying sample sizes (Table 2; Figure S4). Applying the 2 criteria of characterization resulted in the following defining features—cluster 1 (n=6332): no defining feature and considered the healthy cluster; cluster 2 (n=2973): current smoker; cluster 3 (n=719): ever had a cancer diagnosis; cluster 4 (n*=*560): peripheral artery disease; cluster 5 (n=3466): obesity, diabetes, hypertension, and hypertriglyceridemia; cluster 6 (n=528): CHD; cluster 7 (n=27): atrial fibrillation; cluster 8 (n=615): HF; cluster 9 (n=184): renal dysfunction. In addition to these morbidity distributions, we further observed marked demographic and clinical differences between the clusters (Table 1). Participants were younger in cluster 1 (median age, 53 years) than in clusters 6, 7, 8, and 9 (median age, 56–60 years). While the proportion of women was higher in cluster 3 (67%), cluster 4 (69%), cluster 5 (61%), and cluster 8 (72%), members of cluster 6 were mostly men (79%). A higher proportion of Black individuals were found in clusters 5, 8, and 9. Cluster 8 (14%) and cluster 9 (14%) had the highest proportion of participants with incident stroke.

**Table 2.**
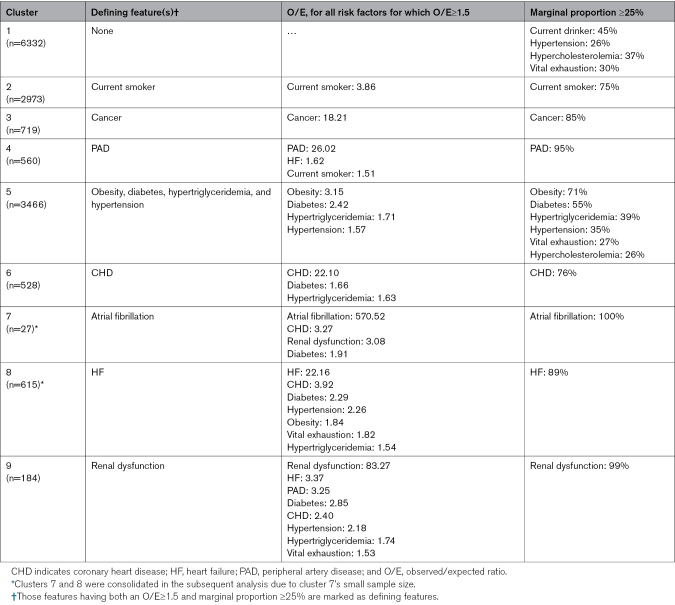
Defining Morbidity Features in the Clusters

### Overall Association of the Clusters With Stroke Incidence

**Figure 1.**
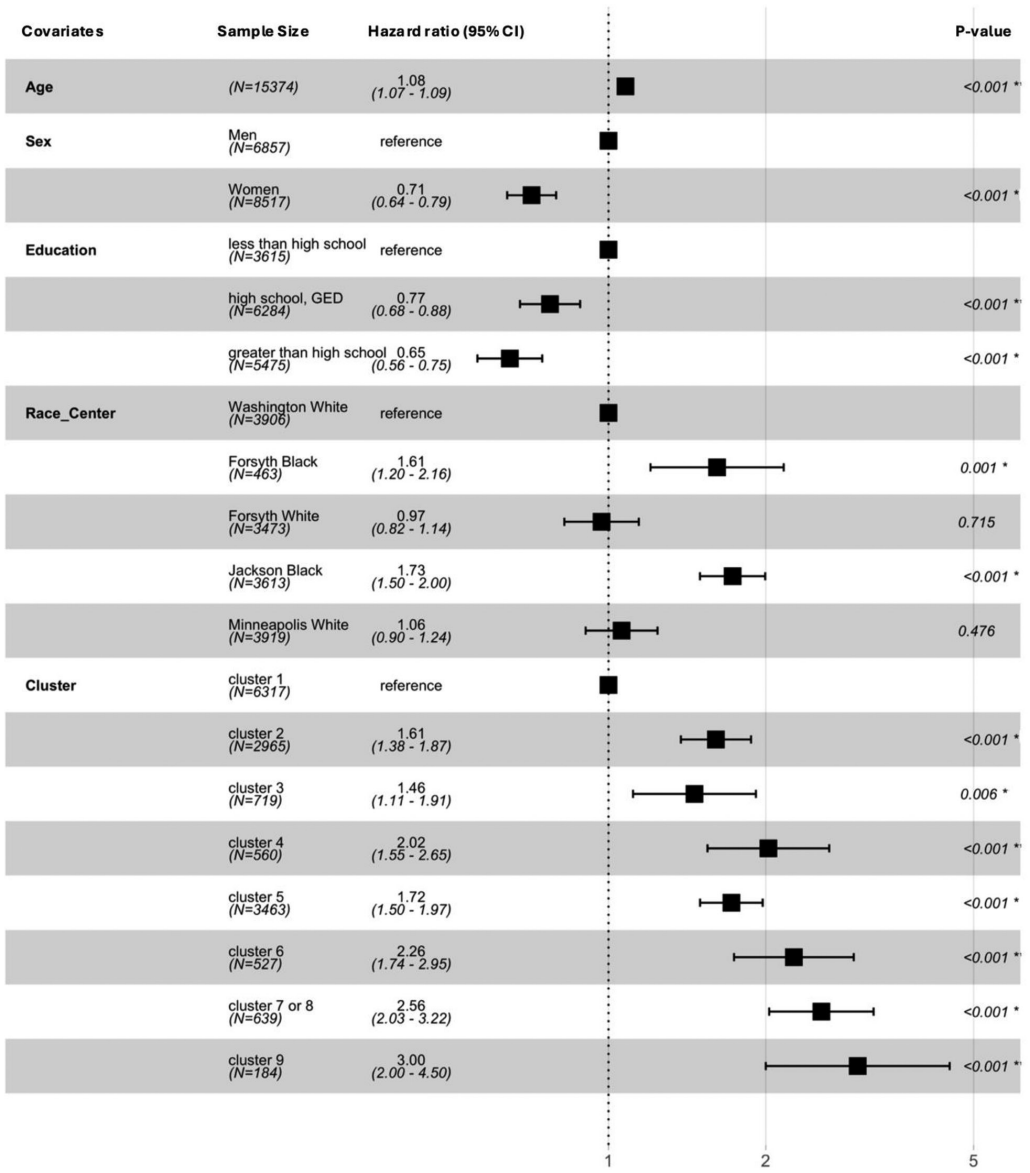
**The association between clusters of morbidities in midlife and overall stroke incidence.** All covariates were entered in the model together. Number of events: 1424; global *P* value (log-rank): 1.7969e−123. Akaike information criterion: 25771.85; concordance index: 0.7. *These *P* values are significant at an α level <0.05; all *P* values are based on the Wald test.

Given the small sample size in cluster 7 (defining feature-atrial fibrillation; n=27), we created a composite cluster 7/8 (atrial fibrillation/HF) in the time-to-event analysis. Participants in each morbidity cluster (clusters 2–9) were more likely to experience an ischemic stroke than those in cluster 1 when accounting for age, sex, education, and race-center (Figure 1). The associations were strongest for cluster 6 (hazard ratio [HR], 2.26 [95% CI, 1.74–2.95]), cluster 7/8 (HR, 2.56 [95% CI, 2.03–3.22]), and cluster 9 (HR, 3.00 [95% CI, 2.00–4.50]), each compared with the healthy group in cluster 1. Independent of the comorbid clusters, stroke risk was higher in Black participants, and lower in women and those with at least a high school education.

### Association of the Clusters With Stroke Incidence, Stratified by Stroke Severity

**Figure 2.**
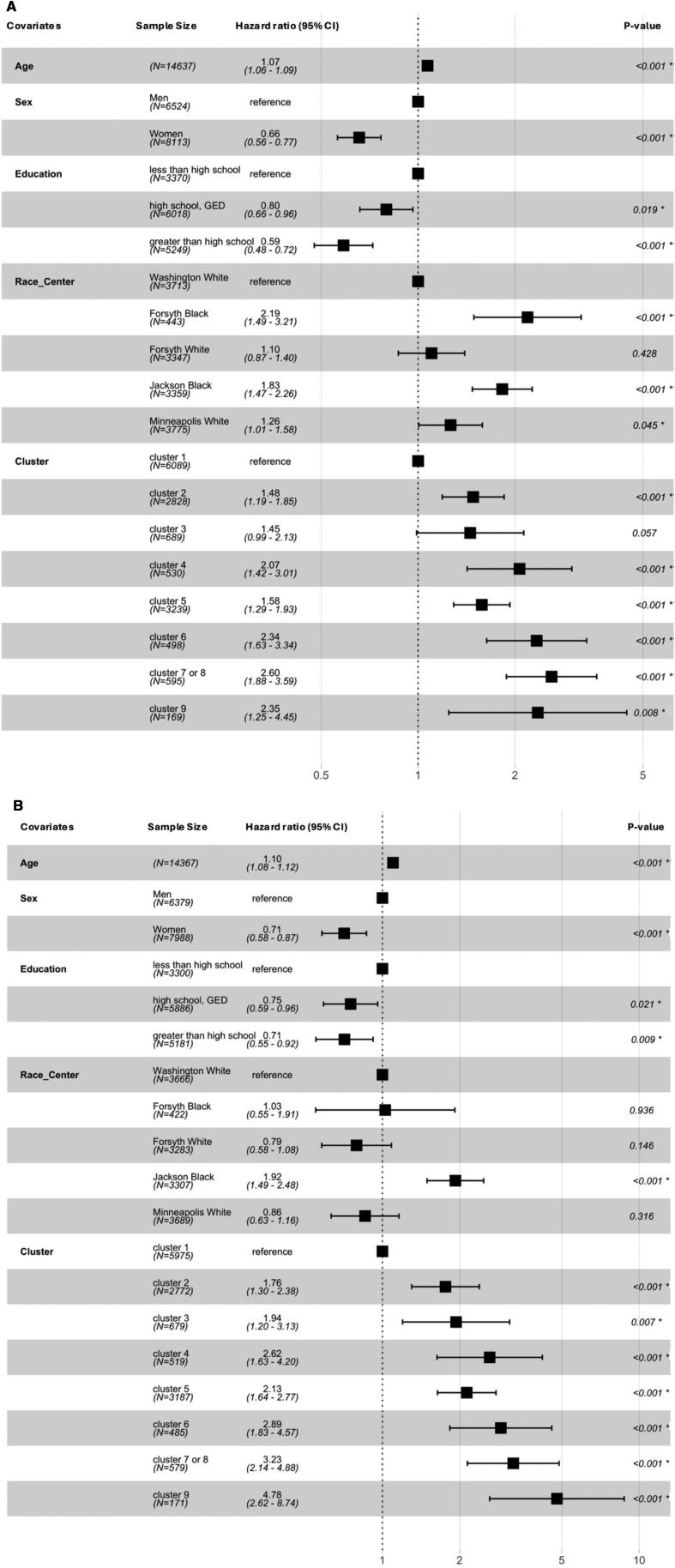
**The association between clusters of morbidities and stroke incidence by severity. A**, Minor-mild stroke (National Institutes of Health Stroke Scale [NIHSS] score ≤5). Number of events: 687; global *P* value (log-rank): 9.2795e−54. AIC: 12515.09; concordance index: 0.7. **B**, Moderate-severe stroke (NIHSS score >5). Number of events: 417; global *P* value (log-rank):1.8305e−55. Akaike information criterion: 7497.52; concordance index: 0.74. All covariates were entered in the model together. *These *P* values are significant at an α level <0.05; all *P* values are based on the Wald test.

When examining stroke by level of severity, the associations of the morbidity clusters were generally stronger for moderate-severe stroke (versus no stroke) than for minor-mild stroke (versus no stroke) incidence (Figure 2A and 2B). The strength of the association with moderate-severe stroke incidence (versus no stroke) was most pronounced for cluster 6 (HR, 2.89 [95% CI, 1.83–4.57], cluster 7/8 (HR, 3.23 [95% CI, 2.14–4.88]), and cluster 9 (HR, 4.78 [95% CI, 2.62–8.74]), each relative to the healthy cluster (cluster 1).

### Interactions Between Demographics and Clusters on Stroke Incidence

When exploring the interaction between the clusters and sex, we observed a significant effect modification by sex for incident moderate-severe stroke but only for cluster 6, defined by CHD (*P*_interaction_=0.01; Table S2). Women (HR, 6.38 [95% CI, 3.20–12.7]) in cluster 6 were at significantly higher risk of moderate-severe (versus no stroke) than were men in cluster 6 (HR, 2.07 [95% CI, 1.13–3.80]), each relative to the healthy cluster groups in the respective sex strata. No significant interaction effect was found for cluster× race, indicating that the clusters’ association with stroke incidence did not significantly differ by race (Table S3). There was, however, an interaction effect between education and cluster 4 for minor-mild stroke incidence (*P*_interaction_=0.04; Table S4). Individuals with less than high school education (HR, 3.18 [95% CI, 1.90–5.33]) in cluster 4 versus cluster 1 were at significantly greater risk for minor-mild stroke (versus no stroke), while the risk associated with cluster 4 for those in the other education groups was not similarly elevated.

### Sensitivity Analysis

When accounting for mortality as a competing risk event, the clusters’ associations with stroke incidence remained significant except for cluster 3 (HR, 1.16 [95% CI, 0.89–1.53]) and cluster 9 (HR, 1.43 [95% CI, 0.94–2.20]; Table S5). The competing risk model still showed that the clusters were more strongly associated with moderate-severe (versus no stroke) than minor-mild (versus no stroke; Tables S6 and S7). This trend also held true for cluster 9, which was significantly associated with moderate-severe stroke (HR, 2.40 [95% CI, 1.29–4.48]) but not with minor-mild stroke incidence (HR, 1.16 [95% CI, 0.60–2.23]).

### Associations of the Clusters With Age at Incident Stroke

When testing the clusters’ association with age of stroke, all morbidity clusters (clusters 2–9) were associated with higher odds of stroke incidence before the age of 70 years (Table 3) when compared with cluster 1. The effect sizes were particularly high for clusters 6 (odds ratio [OR], 3.89 [95% CI, 2.64–5.72]), cluster 7/8 (OR, 3.97 [95% CI, 2.76–5.71]), and cluster 9 (OR, 3.43 [95% CI, 1.87–6.30]). Similar associations with stroke incidence were not observed after the age of 70 years. Only cluster 5, compared with cluster 1, was associated with a greater stroke risk both before the age of 70 years (OR, 2.44 [95% CI, 1.91–3.14]) as well as later in life (OR, 1.20 [95% CI, 1.00–1.43]).

**Table 3.**
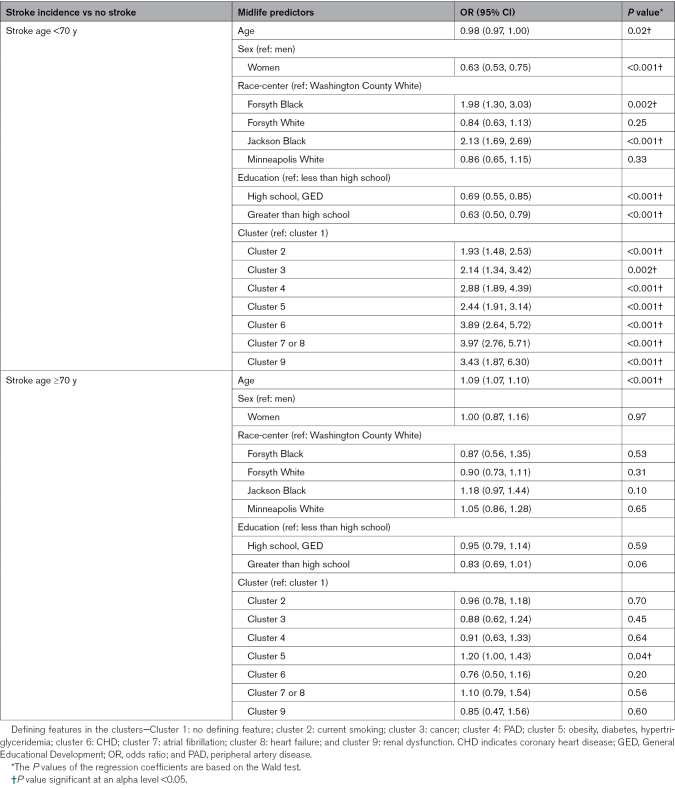
Association Between Morbidity Clusters and Midlife (Age <70 Years) and Late-Life Stroke Incidence (Age ≥70 Years), Each Compared With No Stroke

### Trajectories of the Clusters With Mortality in Late Life

**Figure 3.**
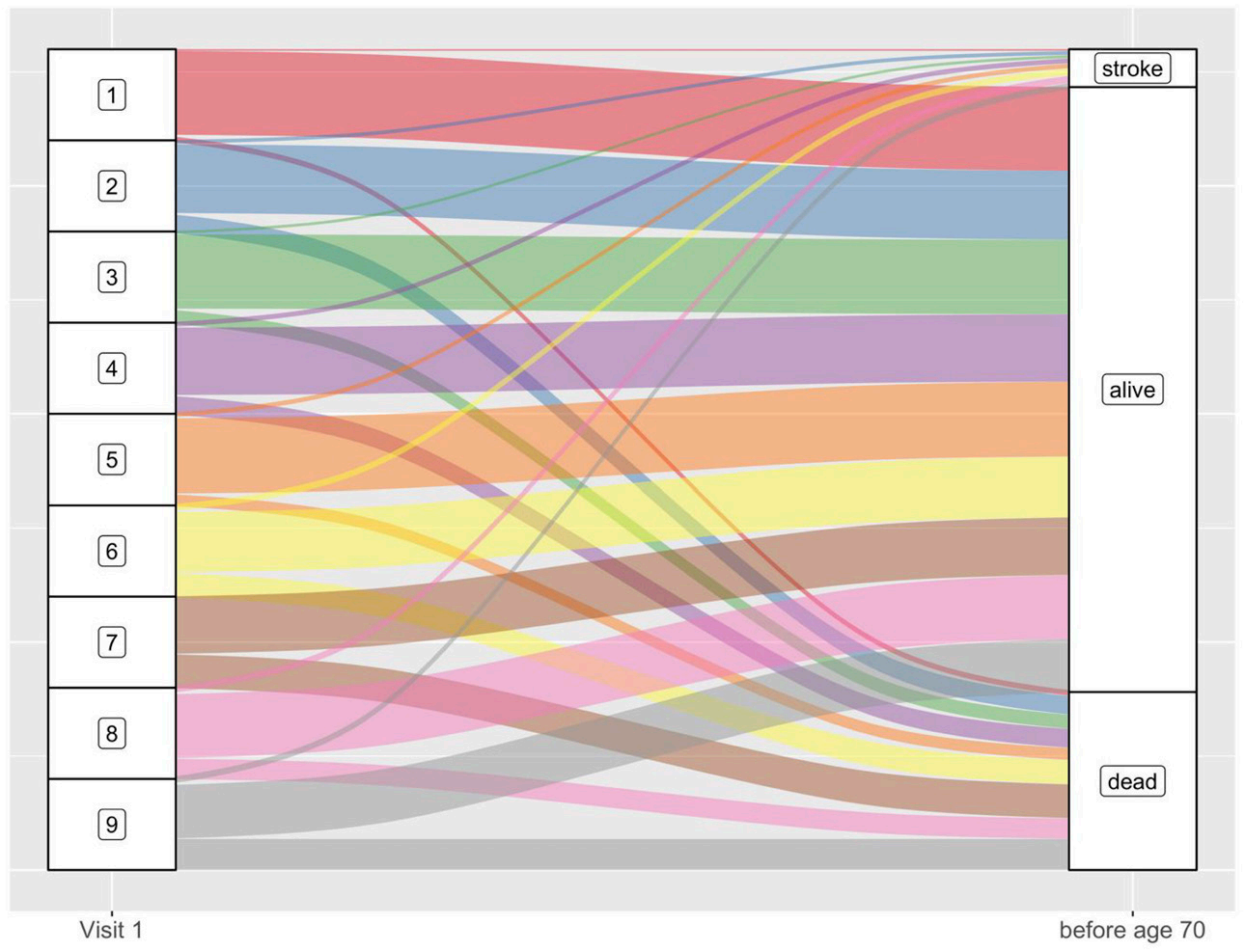
**Cluster trajectories of all individuals at visit 1 based on status prior to the age of 70 years: having previously experienced a stroke, being alive without a stroke, or being deceased without a stroke before the age of 70 years.** Cluster thickness represents the proportion of cases in a cluster having experienced an ischemic stroke, being alive without stroke, or being dead by the age of 70 years. Defining features in the clusters—Cluster 1: no defining feature; cluster 2: current smoking, cluster 3: cancer; cluster 4: peripheral artery disease; cluster 5: obesity, diabetes, hypertriglyceridemia; cluster 6: coronary heart disease; cluster 7: atrial fibrillation; cluster 8: heart failure; and cluster 9: renal dysfunction. *Number of participants (% of that cluster) censored before the age of 70 years by clusters—cluster 1: n=111 (1.8%); cluster 2: n=58 (2.0%); cluster 3: n=8 (1.11%); cluster 4: n=6 (1.1%); cluster 5: n=32 (0.9%); cluster 6: n=2 (0.4%); cluster 7: n=0 (0%); cluster 8: n=6 (1.0%); and cluster 9: n=2 (1.1%). These participants were excluded from the alluvial plot.

To better understand the proportion of participants within each cluster who experienced a stroke before the age of 70 years, were alive without a stroke, and were deceased without a stroke by the age of 70 years, we created an alluvial plot (Figure 3). Mortality significantly varied between the clusters. At least every fifth participant (>20%) in clusters 6 to 9 was deceased without having experienced a stroke before the age of 70 years. Cluster 1 was the only cluster where >90% were still alive at the age of 70 years (Table S8).

## Discussion

In this community-based study, all midlife morbidity clusters were associated with a higher stroke incidence when compared with a cluster of individuals with a healthier risk profile. The association of the clusters was stronger for moderate-severe than for minor-mild stroke. We furthermore showed that, except for cluster 5 defined by obesity, hypertension, diabetes, and hypertriglyceridemia, all midlife morbidity clusters were only associated with stroke before, but not with incident stroke after the age of 70 years. This may be explained by a high mid- to late-life mortality rate in the clusters. At least every fifth participant in the highest morbidity clusters (6–9) was deceased without stroke by the age of 70 years.

Our current understanding is that chronic morbidities do not co-occur at random in an individual, but that they cluster based on specific patterns where they interact with each other, ultimately increasing the risk of negative events beyond the sum of the risk of each risk factor or disease.^14^ Capturing this complexity at a population level is challenging.^31^ Cluster analysis has become a tool to better understand the co-occurrence of chronic conditions.^9^ More recent studies have demonstrated that cluster analysis can be used to group individuals with similar clinical patterns into meaningful clusters, which would allow for a more individual-centered care approach.^9,10^ This study confirmed this, showing that meaningful clusters from as early as midlife can be derived from a large multiracial community.

The strength of the observed association with stroke incidence significantly varied by cluster assignment in midlife and was high in individuals allocated to clusters with CHD and atrial fibrillation/HF as defining features. However, the presence of these defining conditions was only the tip of the iceberg, given that individuals in these clusters were at higher risk for multiple other morbidities. For instance, individuals allocated to cluster 8, defined by HF, were also >3× as likely to have CHD and more than twice as likely to have diabetes and hypertension compared with the overall population. It is likely that HF in combination with other specified morbidities significantly heightened the risk for stroke through previously shown pathophysiological mechanisms.^32,33^ One is the formation of thrombi in the dilated, hypokinetic left ventricle and left atrium.^34,35^ Indirect mechanisms include shared risk factors of stroke, including atherosclerosis and chronic inflammation.^36^

The association with stroke incidence and severity was, however, highest among those individuals allocated to cluster 9 with renal dysfunction as the defining feature and with a significant overrepresentation of HF, peripheral artery disease, diabetes, CHD, and hypertension (observed/expected ratio >2). The co-occurrence of these comorbid risk factors in cluster 9 can be explained by shared complex and multifactorial mechanistic pathways, inherently increasing the risk of stroke.^37^ These include but are not limited to (1) endothelial dysfunction and damage to the blood vessels caused by chronic inflammation which can result in the formation of atherosclerotic plaques, (2) alterations in the blood clotting system, or (3) accumulation of waste products in the blood in the form of uremia which can significantly damage the blood vessels. In line with our findings, a recent AHA presidential advisory has highlighted the multiorgan consequences of cardiovascular-kidney-metabolic syndrome, leading to high incidence rates of cardiovascular diseases and cardiovascular mortality, especially among socially disadvantaged groups.^38^ Particularly among Black individuals, the role of kidney dysfunction has long been recognized as a significant risk factor for cardiovascular events.^39^ Using a disease-centered hypothesis-driven approach, clinical risk scores such as PREVENT,^40^ which includes kidney function as a critical component, have been developed to predict the risk of cardiovascular events. Instead of asking: Which risk scores increase the risk of cardiovascular events?, this study asked: Who shares certain morbidity profiles in the population that are associated with an increased risk of cardiovascular events? We think that this individual-centered data-driven approach, which focuses on shared patterns of morbidities that are not readily apparent when examining individual conditions separately, may provide novel insights into cardiovascular risk stratification in populations. As a result, more personalized treatment plans may be developed by designing interventions for specific subgroups with similar morbidity phenotypes.

Among the risk factors we evaluated, smoking, obesity, diabetes, and hypertension were more prevalent than were other risk factors in midlife, and we identified them as defining features in the 2 largest morbidity clusters. While the defining conditions in cluster 5 (obesity, diabetes, hypertension, and hypertriglyceridemia) each individually increase the stroke risk, previous evidence has shown that their combined presence further potentiates it, leading to amplified risks.^6^ Studies have also highlighted that hyperglycemia and metabolic changes can lead to impaired microvascular circulation via inflammatory pathways and microvascular damage to the inner lining of the blood vessels.^41^ High sustained blood pressure can exacerbate this cascade by causing the blood vessels to weaken and narrow, which can result in altered perfusion in the brain.^42^

There may be several reasons why midlife morbidity clusters were associated with stroke incidence before the age of 70 years, but only cluster 5, defined by diabetes, hypertension, hypertriglyceridemia, and obesity, was also associated with stroke incidence afterwards. One possibility is that early onset and prolonged exposure to diseases have amplified the cumulative damage inflicted on the cerebrovascular system, which in turn increased the risk of stroke events at an earlier age. The clusters’ association with earlier, but not later stroke incidence, may also be due to a high late-life mortality rate, leading to selective attrition (and death) of adults with high midlife comorbidity, thus not giving them an opportunity to have strokes in older age; the evaluation of incident stroke in this study also makes it less likely that those with significant comorbidity in midlife experienced their first stroke only after the age of 70 years. Supporting this hypothesis, we did find that the mortality before the age of 70 years was elevated in clusters defined by CHD, HF, atrial fibrillation, and renal dysfunction (>20%), which is consistent with other reports. Previous studies have shown that kidney disease, the defining feature in cluster 9, is associated with a range of cardiovascular diseases and their progression via mechanisms of inflammation, calcification, uremic toxins, and coagulation, which increases mortality risk.^43^ The rise in mortality in cluster 9, defined by renal dysfunction, may furthermore be explained by multiple underlying risk factors in midlife, which made individuals more vulnerable to cardiovascular events at a younger age.^37^ All these highly morbid clusters include individuals not only with comorbidities in midlife but also already with end-organ injury, suggesting a much longer exposure to the risk factors, likely for years before the baseline study visit.

The study’s findings furthermore showed that participants with lower education and Black individuals were more likely to suffer from moderate-severe strokes, independent of the morbidity clusters. The fact that we find this result even after adjustment for these morbidity clusters emphasizes that disparities in socioeconomic and clinical care may explain these differences, and similar findings have been reported elsewhere.^44,45^ We also observed that women in cluster 6, defined by CHD, were more likely to experience moderate-severe stroke incidence, although most individuals in this cluster were men (79%; Table 1). Previous evidence has shown that women suffer from CHD at a later age with a greater number of cardiovascular risk factors, ultimately increasing mortality risk.^46,47^ It is possible that the cumulative impact of older age, higher cardiovascular burden, and postmenopause changes explains the higher risk for moderate-severe stroke incidence in women. We note, however, that some of the qualitative differences in risk for minor-mild stroke versus moderate-severe stroke may partly reflect smaller sample sizes and imprecision in our estimates, rather than true differences in risk by stroke severity.

The study’s strength is that the analysis used an individual-centered clustering analysis that allocates participants based on their morbidity profile into clusters with similar medical histories. To model these clusters, we used the HCPC function as part of the FactoMineR package, which is a well-established computational library and has frequently been used in research studies before.^30^ This approach allowed us to characterize the co-occurrence of more prevalent risk factors, such as hypertension, and less prevalent, more severe medical conditions, such as HF, in the clusters and defined groups based on combinations of characteristics rather than prespecifying the risk associated with each separate comorbidity. A consequence of this approach, however, is that some risk factors may be common in multiple clusters, although not meeting our defining feature criteria as described above. For instance, hypertension, an often coexisting comorbid risk factor and therefore not surprising, was more prevalent in clusters 5, 8, and 9 than in the overall population, and all these clusters were associated with a greater stroke risk (Table 2). One could therefore conclude that the high prevalence of hypertension as a risk factor alone in those clusters could explain the greater stroke risk when compared with the healthier cluster 1. However, we note that a significant proportion of participants with hypertension (26%) from the overall population were also allocated to cluster 1, serving as the reference cluster in the analysis (Table 2; Figure 1), suggesting that it isn’t the presence of hypertension alone that drives the risk in the other clusters. Instead, the findings illustrate that a higher than expected prevalence of hypertension in combination with diabetes and obesity (cluster 5), with HF (cluster 8), and with renal dysfunction (cluster 9) increases the risk of stroke between mid- and late-life. Again, it is worth emphasizing that cluster analyses are abstraction tools that give us great new insights into the population’s shared morbidity structure. They are, however, less suitable to understand the full spectrum of individual conditions in subpopulations. When assessing the clusters’ association with stroke, it is therefore necessary to focus on the clusters with the defining features instead of only interpreting the defining features themselves.

The clinical advantages of employing an individual-centered clustering analysis instead of a more standard disease-centered risk approach in a population are multifaceted. First, we can quantify the risk of new morbidities occurring in clustered subpopulations with existing morbidities as defining features. For instance, this cluster analysis showed that individuals in cluster 6 with CHD as a defining feature were also 66% more likely to have diabetes (Table 2). From a medical perspective, tight clinical monitoring of diabetes in this subpopulation might therefore be beneficial. Second, as chronic conditions are on the rise, polypharmacy has become a pressing issue, whereby the risk of drug-drug interactions can cause severe adverse events. Understanding the interplay between the cluster’s morbidities might help to optimize medication management in subpopulations and increase treatment efficacy. Third, cluster analysis can also inform about shared underlying mechanisms of morbidities in the subgroups, which in turn could accelerate the development of more targeted therapies. For instance, to reduce the risk of stroke for individuals in cluster 6, new therapeutics might not only consider CHD as the primary target of intervention but would also aim for the underlying mechanistic pathway between CHD and diabetes and hypertriglyceridemia.

The study also has several limitations. First, stroke severity was not prospectively assessed but was based on hospitalization records. However, the information was collected by trained physicians, experienced in evaluations of patients with stroke and specially trained to collect data on stroke severity based on a validated algorithm using the NIHSS, which captures a range of neurological deficits after stroke and is strongly associated with infarct size, another important indicator of severity.^48^ Furthermore, previous findings have demonstrated that hospital chart-based retrospective assessment of stroke severity in ARIC is feasible and reliable.^18,19^ Second, although the ARIC cohort includes a more diverse population than many related studies, only Black and White participants were included in our analysis, given the low proportions of other race groups during recruitment. As a result, the clusters may be different when including individuals from other races and ethnicities. Third, cluster 7 (atrial fibrillation) included only 27 individuals. For the time-to-event analysis, we merged this cluster with cluster 8 (HF), which may obscure AF-specific stroke risk, especially since AF is a strong standalone stroke predictor^49^; thus, the risk association for this group might actually be diluted if compared with a theoretical group evaluating atrial fibrillation alone, if numbers allowed. Fourth, our cluster definitions rely on a data-driven, unsupervised approach rather than hypothesis-driven models. We define clusters using the criteria observed-to-expected ratio and marginal proportion, methods common in multimorbidity research, but acknowledge that alternative clustering techniques or cohorts might yield different groupings. To compare findings across different clustering approaches and different cohort populations, we think it is important to characterize clusters from different populations and to derive defining morbidity features from them based on standardized criteria. Replicating our analysis in other data sets would help distinguish methodological variation from clinical specificity in stroke outcomes across clusters.

This study emphasizes that all morbidity clusters, when compared with a healthier risk profile, were associated with ischemic stroke incidence and with incident moderate to severe stroke. Moreover, this study demonstrates that cluster analysis may be a valuable tool to characterize morbidities in midlife to identify subgroups who are at greater risk for stroke.

## Article Information

### Acknowledgements

The authors thank the staff and participants of the ARIC study (Atherosclerosis Risk in Communities) for their important contributions.

### Sources of Funding

The ARIC study is performed as a collaborative study supported by National Heart, Lung, and Blood Institute contracts 75N92022D00001, 75N92022D00002, 75N92022D00003, 75N92022D00004, and 75N92022D00005. Drs Egle, Groechel, and Gottesman were supported by the National Institute of Neurological Disorders and Stroke Intramural Research Program.

### Disclosures

Dr Johansen reports grants from the American Heart Association; compensation from the American Neurological Association for other services; and grants from the National Institutes of Health. The other authors report no conflicts.

### Supplemental Material

Figures S1–S4

Tables S1–S8

STROBE Checklist

## Supplementary Material


