# Supplementary material for: Role of Morbidity Clusters in Midlife on Ischemic Stroke Incidence and Severity: The ARIC Study
Source: Stroke. 2025 Aug 20;56(10):2928–41. doi: 10.1161/STROKEAHA.124.049496 (PMC12447826; doi:10.1161/STROKEAHA.124.049496)
Supplement: Supplementary file 1 [file str-56-2928-s001.pdf]

SUPPLEMENTARY MATERIALS

Figure S1. Flowchart showing the number of participants (N= 15,404) included in the hierarchical clustering analysis.

Figure S2. Overview of the cluster analysis pipeline

Figure S3. Statistical Overview about the Multiple Correspondence Analysis and Hierarchical Cluster Analysis

Figure S4. Hierarchical clustering tree showing the 9 clusters on the first (Dim 1) and second dimension (Dim 2) derived from the multiple correspondence analysis

Table S1. Key differences between unsupervised and supervised machine-learning models

Table S2. Association between morbidity clusters and incident stroke stratified by sex

Table S3. Association between morbidity clusters and incident stroke stratified by educational attainment

Table S4. Association between morbidity clusters and incident stroke stratified by race

Table S5. Association between the clusters and stroke incidence while accounting for mortality as a competing event.

Table S6. Association between the clusters and minor-mild stroke incidence while accounting for mortality as a competing event.

Table S7. Association between the clusters and moderate-severe stroke incidence while accounting for mortality as a competing event.

Table S8. The proportion of participants by clusters still alive by age 70, deceased by age 70 or censored by age 70.

Figure S1. Flowchart showing the number of participants (*N*= 15,404) included in the hierarchical clustering analysis.

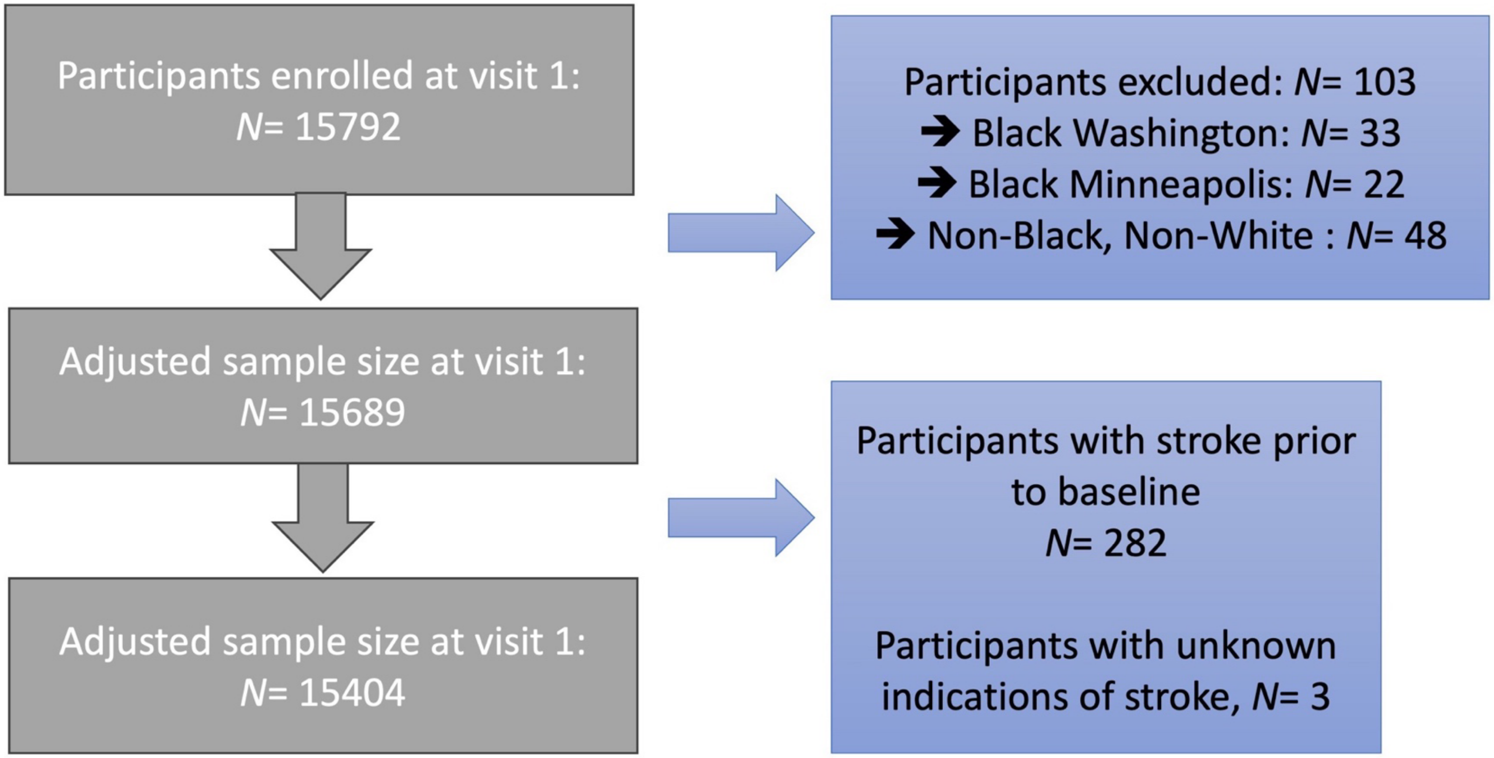

Figure S2. Overview of the cluster analysis pipeline

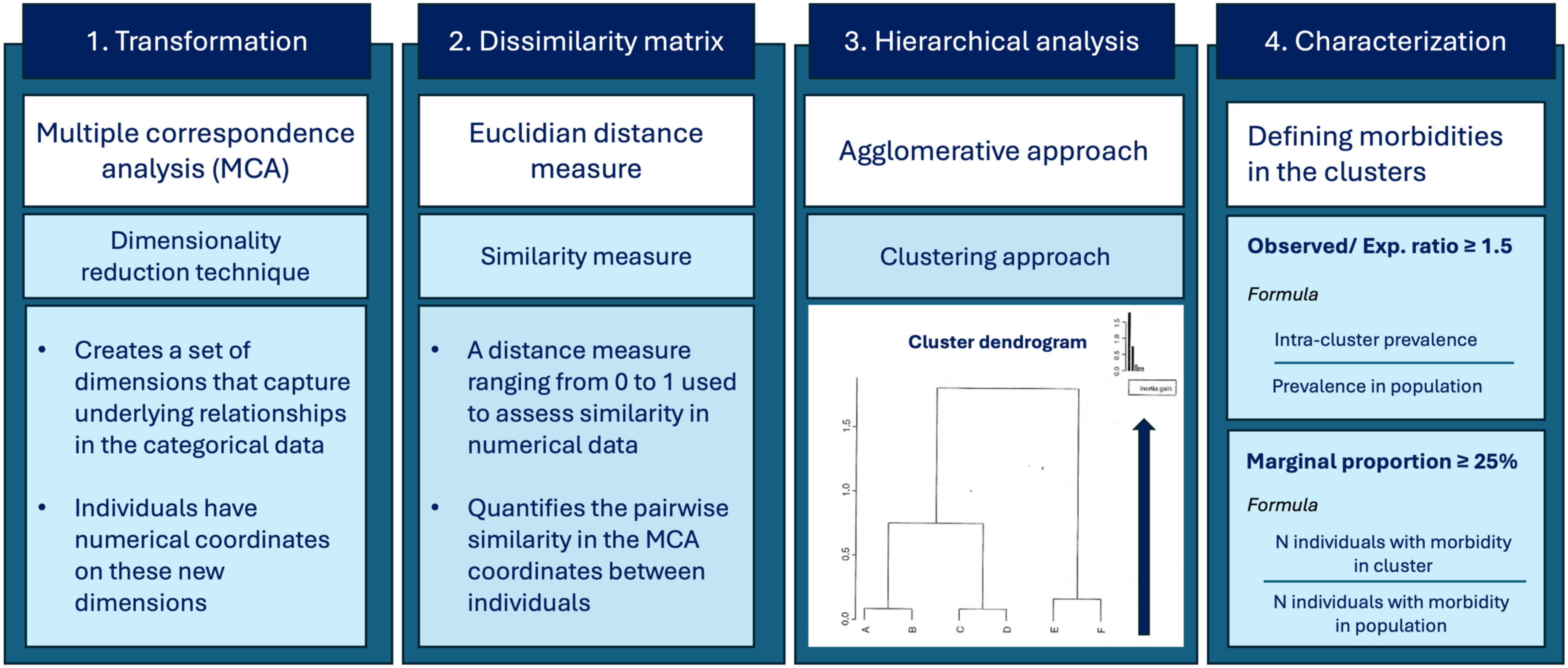

Figure S3. Statistical Overview about the Multiple Correspondence Analysis and Hierarchical Cluster Analysis

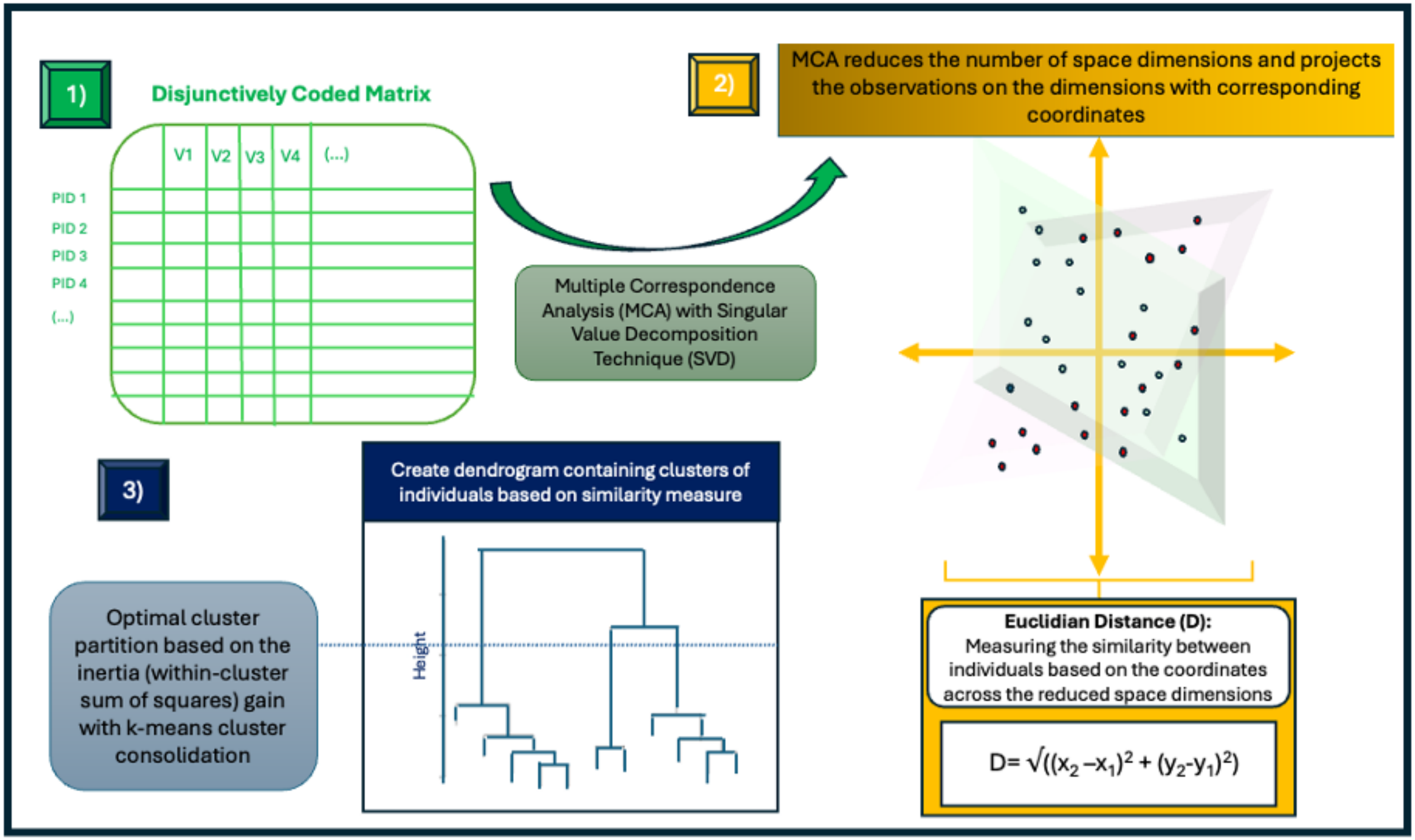

MCA- multiple correspondence analysis; SVD- singular value decomposition; D- Euclidian distance; inertia- within-cluster sum of squares, PID- participant ID, V- variable

Figure S4. Hierarchical clustering tree showing the 9 clusters on the first (Dim 1) and second dimension (Dim 2) derived from the multiple correspondence analysis

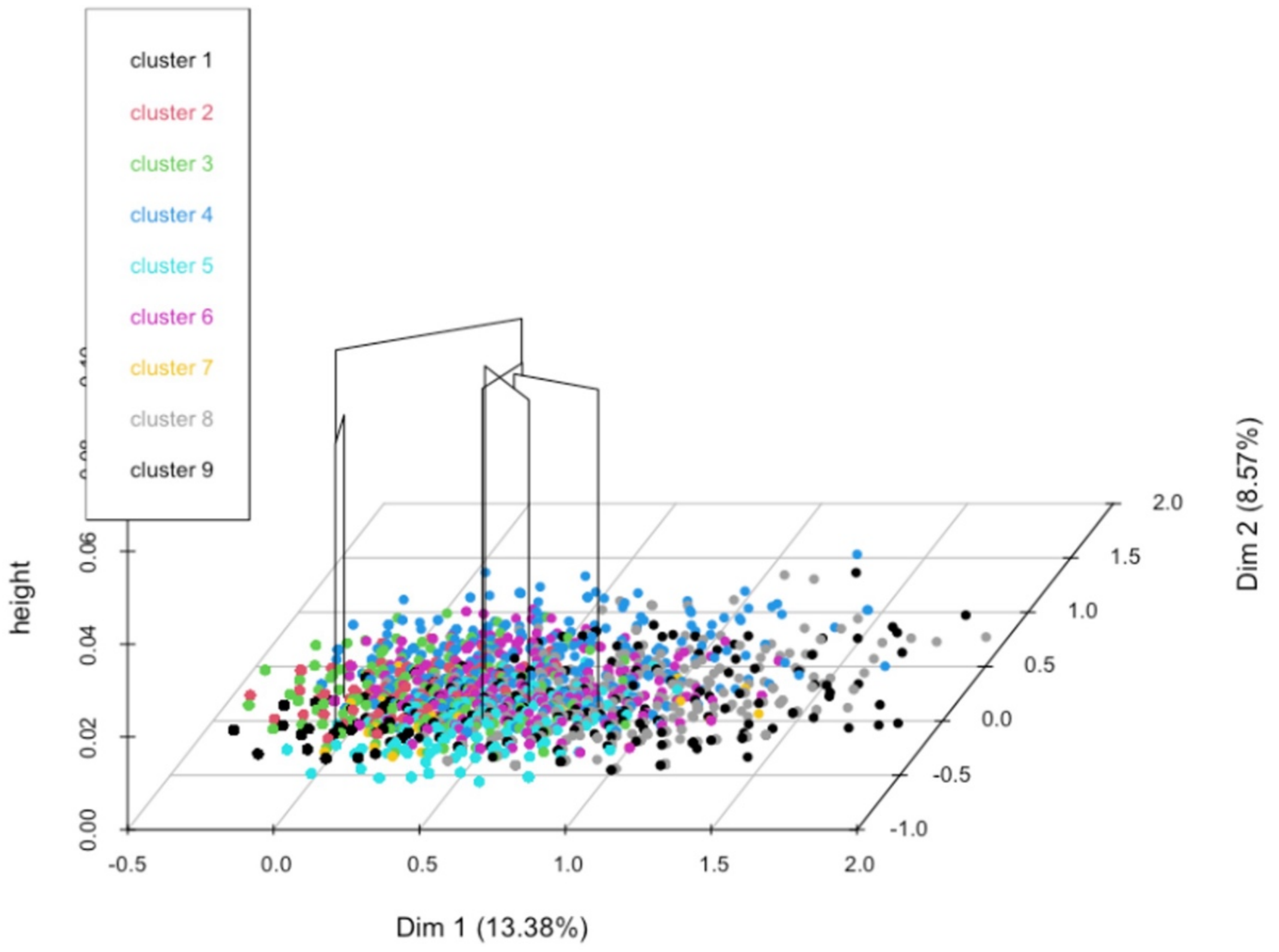

Table S1. Key differences between unsupervised and supervised machine-learning models

**Key Features**	**Unsupervised machine-learning model**	**Supervised machine-learning model**
Data structure	Unlabeled data	Labeled data
User input	Only input data is provided	Outcome measure prediction
Aim	Identification of hidden patterns in the data	Training a model to predict the outcome
User Control	No supervision	External supervision
Methods	Clustering and Transformation methods	Classification, Regression models

Table S2. Association between morbidity clusters and incident stroke stratified by sex

		Overall Stroke		Minor-Mild Stroke		Moderate-Severe Stroke	
Overall, with cluster group as main effect	Cluster	HR[1]	95% CI[1]	HR[1]	95% CI[1]	HR[1]	95% CI[1]
	1	1 (Ref)		1 (Ref)		1 (Ref)	
	2	1.61	1.38, 1.87	1.48	1.19, 1.85	1.76	1.30, 2.38
	3	1.46	1.11, 1.91	1.45	0.99, 2.13	1.94	1.20, 3.13
	4	2.02	1.55, 2.65	2.07	1.42, 3.01	2.62	1.63, 4.20
	5	1.72	1.50, 1.97	1.58	1.29, 1.93	2.13	1.64, 2.77
	6	2.26	1.74, 2.95	2.34	1.63, 3.34	2.89[#]	1.83, 4.57
	7 or 8	2.56	2.03, 3.22	2.60	1.88, 3.59	3.23	2.14, 4.88
	9	3.00	2.00, 4.50	2.35	1.25, 4.45	4.78	2.62, 8.74
Men only	1	1 (Ref)		1 (Ref)		1 (Ref)	
	2	1.50	1.21, 1.86	1.27	0.94, 1.72	1.60	1.06, 2.43
	3	1.50	0.98, 2.28	1.21	0.65, 2.25	1.64	0.75, 3.62
	4	2.70	1.77, 4.12	2.33	1.29, 4.23	3.41	1.68, 6.92
	5	1.69	1.38, 2.07	1.44	1.08, 1.91	2.04	1.41, 2.97
	6	1.99	1.45, 2.74	2.09	1.39, 3.14	2.07	1.13, 3.80
	7 or 8	3.16	2.19, 4.54	3.39	2.13, 5.40	3.84	2.05, 7.20
	9	3.19	1.78, 5.71	0.95	0.23, 3.85	5.80	2.63, 12.8
Women only	1	1 (Ref)		1 (Ref)		1 (Ref)	
	2	1.71	1.37, 2.13	1.78	1.28, 2.46	1.92	1.23, 3.00
	3	1.45	1.02, 2.06	1.73	1.06, 2.85	2.15	1.16, 3.98
	4	1.73	1.22, 2.45	2.03	1.25, 3.31	2.23	1.18, 4.21
	5	1.72	1.42, 2.09	1.74	1.30, 2.32	2.23	1.54, 3.25
	6	3.37	2.10, 5.42	2.92	1.35, 6.32	6.38	3.20, 12.7
	7 or 8	2.30	1.71, 3.11	2.25	1.44, 3.53	3.05	1.76, 5.28

	9	2.80	1.59, 4.92	3.91	1.88, 8.12	3.71	1.46, 9.41

[1]*HR- Hazard ratio, CI- confidence interval*
[#] Interaction effect cluster * sex was statistically significant ($p < 0.05$) in the overall analysis

Table S3. Association between morbidity clusters and incident stroke stratified by race

		Overall Stroke		Minor-Mild Stroke		Moderate-Severe Stroke	
Overall, with cluster group as main effect	Cluster	HR[1]	95% CI[1]	HR[1]	95% CI[1]	HR[1]	95% CI[1]
	1	1 (Ref)		1 (Ref)		1 (Ref)	
	2	1.61	1.38, 1.87	1.48	1.19, 1.85	1.76	1.30, 2.38
	3	1.46	1.11, 1.91	1.45	0.99, 2.13	1.94	1.20, 3.13
	4	2.02	1.55, 2.65	2.07	1.42, 3.01	2.62	1.63, 4.20
	5	1.72	1.50, 1.97	1.58	1.29, 1.93	2.13	1.64, 2.77
	6	2.26	1.74, 2.95	2.34	1.63, 3.34	2.89	1.83, 4.57
	7 or 8	2.56	2.03, 3.22	2.60	1.88, 3.59	3.23	2.14, 4.88
	9	3.00	2.00, 4.50	2.35	1.25, 4.45	4.78	2.62, 8.74
Black participants only**	1	1 (Ref)		1 (Ref)		1 (Ref)	
	2	1.50	1.14, 1.97	1.41	0.94, 2.12	1.34	0.81, 2.23
	3	2.46	1.38, 4.39	1.97	0.78, 4.96	2.87	1.11, 7.39
	4	2.22	1.44, 3.41	2.72	1.54, 4.80	2.58	1.23, 5.42
	5	1.74	1.37, 2.20	1.63	1.15, 2.32	2.15	1.41, 3.26
	6	2.80	1.68, 4.68	3.05	1.50, 6.21	3.36	1.48, 7.62
	7 or 8	2.92	2.06, 4.14	3.33	2.05, 5.40	2.72	1.42, 5.23
	9	2.70	1.36, 5.37	2.33	0.84, 6.50	3.26	1.14, 9.30
White participants only**	1	1 (Ref)		1 (Ref)		1 (Ref)	
	2	1.71	1.41, 2.06	1.61	1.24, 2.10	2.02	1.38, 2.94
	3	1.29	0.95, 1.76	1.36	0.89, 2.08	1.73	0.99, 3.02
	4	1.91	1.35, 2.71	1.69	1.01, 2.83	2.62	1.42, 4.84
	5	1.75	1.47, 2.08	1.56	1.21, 2.00	2.19	1.55, 3.08
	6	2.14	1.57, 2.92	2.12	1.40, 3.22	2.90	1.67, 5.04

	7 or 8	2.26	1.64, 3.11	2.07	1.30, 3.30	3.56	2.09, 6.06
	9	3.22	1.94, 5.32	2.39	1.06, 5.42	6.47	3.10, 13.5

[1]HR- Hazard ratio, CI- confidence interval
**The following covariates were included in the models but not shown in the table: age, education, race-center, and sex*
***The following covariates were included in the race-stratified models but not shown in the table: age, education, and sex*

Table S4. Association between morbidity clusters and incident stroke stratified by education

		Overall Stroke		Minor-Mild Stroke		Moderate-Severe Stroke	
Overall, with cluster group as main effect	Cluster	HR[1]	95% CI[1]	HR[1]	95% CI[1]	HR[1]	95% CI[1]
	1	1 (Ref)		1 (Ref)		1 (Ref)	
	2	1.61	1.38, 1.87	1.48	1.19, 1.85	1.76	1.30, 2.38
	3	1.46	1.11, 1.91	1.45	0.99, 2.13	1.94	1.20, 3.13
	4	2.02	1.55, 2.65	2.07[#]	1.42, 3.01	2.62	1.63, 4.20
	5	1.72	1.50, 1.97	1.58	1.29, 1.93	2.13	1.64, 2.77
	6	2.26	1.74, 2.95	2.34	1.63, 3.34	2.89	1.83, 4.57
	7 or 8	2.56	2.03, 3.22	2.60	1.88, 3.59	3.23	2.14, 4.88
	9	3.00	2.00, 4.50	2.35	1.25, 4.45	4.78	2.62, 8.74
Less than high school	1	1 (Ref)		1 (Ref)		1 (Ref)	
	2	1.38	1.03, 1.83	1.22	0.81, 1.83	1.36	0.81, 2.28
	3	1.11	0.59, 2.07	1.00	0.40, 2.51	1.57	0.61, 4.06
	4	2.36	1.57, 3.54	3.18	1.90, 5.33	1.73	0.76, 3.94
	5	1.53	1.18, 1.97	1.36	0.94, 1.98	1.56	0.99, 2.45
	6	2.08	1.34, 3.24	1.65	0.85, 3.19	3.17	1.62, 6.21
	7 or 8	2.73	1.91, 3.90	2.60	1.55, 4.37	2.86	1.53, 5.33
	9	2.92	1.47, 5.80	3.14	1.24, 7.95	2.55	0.77, 8.43
High school or GED	1	1 (Ref)		1 (Ref)		1 (Ref)	
	2	1.64	1.28, 2.09	1.67	1.19, 2.35	1.59	0.95, 2.65
	3	1.62	1.08, 2.41	1.87	1.09, 3.19	1.86	0.87, 3.99
	4	1.74	1.08, 2.79	1.31	0.61, 2.84	3.05	1.47, 6.32
	5	1.86	1.49, 2.31	1.97	1.44, 2.69	2.10	1.35, 3.25
	6	2.53	1.64, 3.89	2.73	1.54, 4.83	2.81	1.25, 6.32
	7 or 8	1.96	1.28, 2.98	2.58	1.51, 4.42	1.79	0.75, 4.23
	9	3.31	1.74, 6.28	1.36	0.33, 5.52	7.41	3.11, 17.6

Above high school	1	1 (Ref)		1 (Ref)		1 (Ref)	
	2	1.78	1.34, 2.37	1.50	0.98, 2.29	2.43	1.40, 4.24
	3	1.51	0.96, 2.39	1.29	0.65, 2.58	2.30	1.02, 5.19
	4	1.64	0.91, 2.96	1.06	0.39, 2.90	2.99	1.16, 7.70
	5	1.73	1.35, 2.22	1.38	0.94, 2.03	2.83	1.77, 4.53
	6	2.10	1.25, 3.53	2.78	1.47, 5.27	2.11	0.74, 5.99
	7 or 8	2.83	1.77, 4.52	2.42	1.21, 4.85	5.45	2.59, 11.5
	9	2.56	1.12, 5.81	2.35	0.73, 7.52	5.04	1.52, 16.7

[1]*HR- Hazard ratio, CI- confidence interval*

**The following covariates were included in the models but not shown in the table: age, education, race-center, and sex*

***The following covariates were included in the race-stratified models but not shown in the table: age, race-center, and sex*

[#] *Interaction effect cluster * education was statistically significant (p< 0.05) in the overall analysis*

Table S5. Association between the clusters and stroke incidence while accounting for mortality as a competing event.

**Characteristics**	**HR**	**95% CI**
Age	1.04	1.03, 1.05
Sex (ref: men)		
Women	0.85	0.76, 0.95
Race-Center<br>(ref: Washington county White)		
Forsyth Black	1.33	0.99, 1.79
Forsyth White	0.92	0.78, 1.08
Jackson Black	1.52	1.32, 1.76
Minneapolis White	1.01	0.86, 1.18
Education<br>(ref: less than high school)		
high school, GED	0.84	0.74, 0.96
greater than high school	0.75	0.64, 0.86
Cluster (ref: cluster 1)		
2	1.20	1.03, 1.40
3	1.16	0.89, 1.53
4	1.39	1.06, 1.82
5	1.48	1.29, 1.70
6	1.49	1.13, 1.95
7 or 8	1.76	1.40, 2.23
9	1.43	0.94, 2.20

[1] HR = Hazard Ratio, CI = Confidence Interval

all covariates were entered in the model together

Table S6. Association between the clusters and minor-mild stroke incidence while accounting for mortality as a competing event.

**Characteristics**	**HR**	**95% CI**
Age	1.04	1.03, 1.06
Sex (ref: men)		
Women	0.76	0.65, 0.89
Race-Center<br>(ref: Washington county White)		
Forsyth Black	1.85	1.26, 2.72
Forsyth White	1.05	0.83, 1.33
Jackson Black	1.62	1.31, 2.01
Minneapolis White	1.21	0.96, 1.52
Education<br>(ref: less than high school)		
high school, GED	0.86	0.71, 1.05
greater than high school	0.67	0.54, 0.83
Cluster<br>(ref: cluster 1)		
2	1.15	0.93, 1.44
3	1.19	0.81, 1.74
4	1.47	1.01, 2.13
5	1.39	1.14, 1.70
6	1.62	1.12, 2.33
7 or 8	1.86	1.34, 2.58
9	1.16	0.60, 2.23

[1] HR = Hazard Ratio, CI = Confidence Interval

all covariates were entered in the model together

Table S7. Association between the clusters and moderate-severe stroke incidence while accounting for mortality as a competing event.

**Characteristics**	**HR**	**95% CI**
Age	1.07	1.05, 1.09
Sex (ref: men)		
Women	0.83	0.68, 1.02
Race-Center (ref: Washington county White)		
Forsyth Black	0.87	0.47, 1.64
Forsyth White	0.76	0.56, 1.05
Jackson Black	1.71	1.32, 2.20
Minneapolis White	0.82	0.60, 1.11
Education (ref: less than high school)		
high school, GED	0.81	0.63, 1.04
greater than high school	0.81	0.62, 1.05
Cluster (ref: cluster 1)		
2	1.39	1.02, 1.88
3	1.58	0.98, 2.55
4	1.84	1.14, 2.97
5	1.89	1.45, 2.47
6	2.04	1.28, 3.25
7 or 8	2.33	1.54, 3.53
9	2.40	1.29, 4.48

[1] HR = Hazard Ratio, CI = Confidence Interval

all covariates were entered in the model together

Table S8. The proportion of participants by clusters having experienced a stroke before age 70, being alive without a stroke before age 70, deceased before age 70, or censored before age 70.

	**Stroke before age 70**	**Alive without stroke before age 70**	**Deceased without stroke before age 70**	**Censored before age 70**
Cluster 1 (N= 6332)	108 (1.7%)	5725 (90.4%)	388 (6.1%)	111 (1.8%)
Cluster 2 (N= 2973)	120 (4.0%)	2202 (74.1%)	593 (20.0%)	58 (2.0%)
Cluster 3 (N= 719)	22 (3.1%)	580 (80.7%)	109 (15.2%)	8 (1.1%)
Cluster 4 (N= 560)	30 (5.4%)	410 (73.2%)	114 (20.4%)	6 (1.1%)
Cluster 5 (N= 3466)	174 (5.0%)	2815 (81.2%)	445 (12.8%)	32 (0.9%)
Cluster 6 (N= 528)	39 (7.4%)	348 (65.9%)	139 (26.3%)	2 (0.4%)
Cluster 7 (N= 27)	0 (0%)	17 (63.0%)	10 (37.0%)	0 (0%)
Cluster 8 (N= 615)	46 (7.5%)	427 (69.4%)	136 (22.1%)	6 (1.0%)
Cluster 9 (N= 184)	13 (7.1%)	107 (58.2%)	62 (33.7%)	2 (1.1%)
