# Supplementary material for: Role of Morbidity Clusters in Midlife on Ischemic Stroke Incidence and Severity: The ARIC Study
Source: Stroke. 2025 Aug 20;56(10):2928–41. doi: 10.1161/STROKEAHA.124.049496 (PMC12447826; doi:10.1161/STROKEAHA.124.049496)
Supplement: Supplementary file 2 [file str-56-2928-s002.pdf]

**AHA Journals Racial and Ethnic Disparities Reporting Guidelines**			
**Section/Topic**	**Item No.**	**Recommendation**	**Provide page # in text / Confirm that this was "done throughout" / N/A**
**Introduction**			
	1a	Discuss a framework (e.g., conceptual model) for studying race and/or ethnicity in this context	NA
	1b	Discuss social and structural forms of racism and/or bias	NA
**Methods**			
Categorization of race/ethnicity	2a	Describe categorization of race and ethnicity (e.g., self-identification)	p6
	2b	If race and ethnicity are codified by others, be specific in conveying how the categories were attributed	p6
	2c	Describe potential limitations of existing data sources	p6
Terminology			
	3a	Capitalize race and ethnicity terms and use as adjectives rather than nouns (e.g., "Black patients," not "blacks", "White patients," not "whites", etc.)	done throughout
	3b	Describe specific racial and ethnic makeup of smaller population groups; when possible avoid "non-White" or "Other"	p6
	3c	Use accurate terminology: Hispanic and Latino/a/-x are ethnicities; the term "White" is preferred over Caucasian	done throughout
Analyses			
	4a	Provide context and analytical use of race/ethnicity as a covariate in risk-adjustment models	p10
**Results**			
	5a	Avoid statements of causal inference or culpability (e.g., "Black adults did not respond to *x* medication." Instead, "Among Black adults, *x* medication was less effective")	done throughout
**Discussion**			
	6a	Describe the relevant structural and social factors that influence the study question	p18
	6b	Avoid using genetics in isolation to explain social constructs	NA
